# High concentrations of divalent cations isolate monosynaptic inputs from local circuits in the auditory midbrain

**DOI:** 10.3389/fncir.2013.00175

**Published:** 2013-10-29

**Authors:** Shobhana Sivaramakrishnan, Jason Tait Sanchez, Calum Alex Grimsley

**Affiliations:** Department of Anatomy and Neurobiology, Northeast Ohio Medical UniversityRootstown, OH, USA

**Keywords:** high divalents, inferior colliculus, monosynaptic, local circuits, first spike latency

## Abstract

Hierarchical processing of sensory information occurs at multiple levels between the peripheral and central pathway. Different extents of convergence and divergence in top down and bottom up projections makes it difficult to separate the various components activated by a sensory input. In particular, hierarchical processing at sub-cortical levels is little understood. Here we have developed a method to isolate extrinsic inputs to the inferior colliculus (IC), a nucleus in the midbrain region of the auditory system, with extensive ascending and descending convergence. By applying a high concentration of divalent cations (HiDi) locally within the IC, we isolate a HiDi-sensitive from a HiDi-insensitive component of responses evoked by afferent input in brain slices and *in vivo* during a sound stimulus. Our results suggest that the HiDi-sensitive component is a monosynaptic input to the IC, while the HiDi-insensitive component is a local polysynaptic circuit. Monosynaptic inputs have short latencies, rapid rise times, and underlie first spike latencies. Local inputs have variable delays and evoke long-lasting excitation. *In vivo*, local circuits have variable onset times and temporal profiles. Our results suggest that high concentrations of divalent cations should prove to be a widely useful method of isolating extrinsic monosynaptic inputs from local circuits *in vivo*.

## INTRODUCTION

How does the representation of a sensory stimulus in the periphery get transformed into a perceptually reliable code in the cortex? Hierarchical flow of information converts the representation of sensory features extracted at the periphery into central codes. Cortical processing is the target of most hierarchical activity, and it depends to a great extent on sub-cortical structures that provide both driving and modulating components along the information path.

Hierarchical processing occurs at multiple levels of the auditory system. The ascending part of the central auditory system involves extensive convergence and divergence of inputs (Oliver et al., 1997; Cant and Benson, 2003), which can be difficult to untangle. This gives rise to the need to develop methods for isolating extrinsic inputs from local influences. In the auditory cortex, ascending monosynaptic and local intra-cortical influences on cortical responses to acoustic input are separated through a cocktail of GABA_A_ agonists and GABA_B_ antagonists, which isolate thalamocortical input from local inhibitory intracortical circuits (Liu et al., 2007). This method would not work in the midbrain, or between different cortical areas, however, where extrinsic and local pathways consist of a mix of excitatory and inhibitory inputs. We therefore developed a method to separate extrinsic monosynaptic inputs from local circuits in a nucleus that receives extensive ascending and descending projections that include excitatory and inhibitory inputs.

The inferior colliculus (IC), a nucleus in the auditory midbrain, receives massive input convergence from lower auditory nuclei (Oliver et al., 1997; Malmierca et al., 2002) and descending influences through corticofugal projections (Winer, 2005). Extensive local circuitry within and between colliculi connects layers of cells that receive inputs at different frequencies (Oliver et al., 1991; Malmierca et al., 2009; Chandrasekaran et al., 2013). We isolated extrinsic monosynaptic inputs from local circuits by applying a high concentration of divalent cations (HiDi; Frankenhaeuser and Hodgkin, 1957) to IC neurons in brain slices and *in vivo*. Increases in divalent cation concentrations raise firing threshold by increasing surface charge (Hille et al., 1975) and creating positive shifts in the activation kinetics of voltage-gated sodium channels (Campbell and Hille, 1976). Moderate increases in divalent cations are thought to prevent polysynaptic inputs from reaching firing threshold without affecting monosynaptic inputs (Nicholls and Purves, 1970; Byrne et al., 1978; Liao and Walters, 2002; Einum and Buchanan, 2004). Here we describe the use of HiDi in the IC, first by establishing criteria for the separation of monosynaptic from local inputs activated by stimulation of lateral lemniscal afferents in brain slices, and then testing the effects of HiDi *in vivo* on inputs activated by sound.

## MATERIALS AND METHODS

CBA/Ca mice were obtained from Jackson Labs, Bar Harbor, ME, USA or from our in-house breeding colonies. All animal procedures for *in vitro* brain slice and *in vivo recordings* were approved by the Committee for Animal Care and Use at the Northeast Ohio Medical University and conformed to the guidelines for laboratory animal care and use published by the National Institutes for Health.

### BRAIN SLICE RECORDINGS

Brains from 25- to 35-day-old mice were used for slice recordings. The methods are described briefly here since they have been published in detail (Chandrasekaran et al., 2013). Mice were anesthetized with isoflurane and decapitated. The brain was removed and 300 μm thick slices were made through the transverse plane of the IC. For recordings, slices were transferred to a temperature-regulated recording chamber and experiments were carried out at 35°C. The slice was superfused at 2 ml/min with oxygenated (95%O_2_/5%CO_2_) artificial cerebrospinal fluid (ACSF) containing (in millimoles): 130 NaCl, 3 KCl, 2 CaCl_2_, 1.3 MgSO_4_, 1 NaH_2_PO_4_, 26 NaHCO_3_, 25 glucose, pH 7.35.

Whole-cell patch-clamp recordings were made from the central region of the IC. Recordings were made under visual control using an upright microscope (Zeiss Axioskop) fitted with a water immersion objective (×40/NA 0.75) and differential interference optics. Patch pipettes were made from borosilicate glass (Kimax, 1.5 mm O.D.), with resistances of 5–7 MΩ when filled with a recording solution containing, in millimoles: 120 K Gluconate, 10 KCl, 0.2 EGTA, 0.1 CaCl_2_, 4 Mg-ATP, 0.3 Na-GTP, 10 HEPES, 10 phosphocreatine, pH 7.3; free Ca^+^^+^ 90 nM. Series resistances were generally 12–18 MΩ and compensated by 75–80%. A junction potential correction of -11 mV was applied to all voltages; reported resting membrane potentials include this correction. An EPC-10 amplifier and Patchmaster/Fitmaster software (HEKA Elektroniks/Instrutech Corporation) were used respectively for recordings, data collection, and analyses. Origin software (OriginLab) was used for statistical analysis and graphing data. Data are reported from 166 cells in 58 slices.

#### High-divalent solutions

For experiments where a HiDi concentration was used, ACSF was made without NaH_2_PO_4_ and MgCl_2_ was substituted for MgSO_4_ (to prevent precipitation of calcium phosphate or sulfate), and a HiDi concentration reached by increasing the concentrations of CaCl_2_ and MgCl_2_, with a compensatory reduction of the NaCl concentration to balance osmolality. To prevent competitive block of presynaptic Ca^+^^+^ channels and allow normal transmitter release, the ratio of Ca^+^^+^ to Mg^+^^+^ was the same as the control (Byrne et al., 1978; e.g., 2.5 HiDi = 5 mM CaCl_2_, 3.25 mM MgCl_2_; total increase in divalents = 4.95 mM). We tested several HiDi concentrations on intrinsic membrane properties (see Results) to determine an optimal concentration that would isolate monosynaptic from polysynaptic inputs without altering intrinsic firing patterns. HiDi was applied either locally through a second electrode close to the cell (*n* = 26 slices) or bath applied to control for incomplete effects on dendritic branches (*n* = 32 slices). HiDi effects on intrinsic properties and synaptic transmission did not differ significantly between these two application methods (comparison of peak amplitudes of synaptic currents evoked by maximum lemniscal stimulation in control ACSF and HiDi; *t*_87_ = 1.4; *p* = 0.16), suggesting that HiDi application using both methods reached (electrotonically) most of the synapses on the neuron from which recordings were made. For complete washout from HiDi, ACSF was perfused for 5–10 min and intrinsic firing patterns or synaptic responses checked for recovery.

#### Lemniscal stimulation

To restrict the spatial spread of current as much as possible to the immediate vicinity of the stimulating electrode, synaptic activity was evoked by stimulating the lateral lemniscus (LL) with a concentric extracellular bipolar electrode. We used an electrode with a tip diameter of ~100 μm as a way to recruit the largest possible number of axons at maximum current strengths. In some experiments, we switched to a bipolar two-pronged stimulating electrode straddled across the lemniscal tract, to check the response amplitude at maximum stimulus currents. A 100 μm tip diameter would cover a large percentage of the axons in the lemniscal tract, thus it would be expected that minimal stimulation, defined by the recruitment of a single axon, would not be possible. In other experiments we used concentric electrodes with smaller active diameters, but these electrodes did not give us the full range of synaptic activity, suggesting inadequate effective current spread, which would selectively eliminate axons with smaller diameters from our data. With the 100 μm tip diameter, responses at minimal stimulation, which was defined as a 50% failure rate, evoked synaptic currents between 30 and 60 pA, which we estimate to be three to four times the amplitude of a spontaneous miniature synaptic current in IC neurons (Sivaramakrishnan and Oliver, 2006). Thus we think that we were able to isolate a few synapses with minimal stimulation.

The stimulating electrode was placed ventral to the dorsal nucleus of the lateral lemniscus and to the IC, allowing stimulation of LL fibers of passage from lower brainstem nuclei and from the dorsal nucleus of the LL. Stimulus pulses generated by a stimulator (AMPI, Israel) were passed through a constant current isolation unit (A365; WPI, Sarasota, FL, USA) before reaching the stimulating electrode. Biphasic current pulses were used to prevent DC build-up on the LL tract during repeated stimulation, preventing repeated activation of the same LL afferent axon at high stimulus intensities (with a single shock). Minimal currents (<0.5 mA; 0.1–0.3 ms) were those that evoked postsynaptic potentials (PSPs) 50% of the time in 40–50 trials. Maximal currents were those beyond which PSPs did not change in amplitude or duration and were taken to indicate maximum recruitment of LL afferent axons (100% LL activation). Current strengths during stimulus trains were adjusted in each slice to generate the responses needed. Inter-trial intervals were generally >500 ms for single pulse stimulation and at least >2 s for stimulus trains with <10 pulses.

Recording modes were switched between voltage- and current-clamp to record postsynaptic currents (PSCs) and potentials (PSPs) respectively. Holding potentials for PSCs were adjusted for each cell to match the value of the resting membrane potential measured under current-clamp. PSPs and PSCs were averaged from 5 to 10 repeated trials, unless otherwise specified. In some recordings of PSPs under current clamp, we included QX-314 in the recording pipette to block voltage-gated conductances (Mulle et al., 1985). This prevented identification of cells based on their intrinsic firing properties (Sivaramakrishnan and Oliver, 2001), thus we pooled data from different cell types.

#### Statistical tests

For collection of synaptic currents and potentials, trials were repeated several times at low rates (1/5s; 4–10 trials), averaged within a single slice, and then averaged across slices. For measurements of onset latencies we used data from cells in which there was a clear separation between stimulus artifact and PSC onset; i.e., a period of flat baseline before PSC onset. At very high stimulus currents, there were no PSCs that met this criterion. Therefore, the PSC onset was taken as the time of 20% deviation from baseline for all PSCs at all LL currents. PSC and PSP durations were measured at 10% above baseline. Integrals of PSCs and PSPs were measured as the area under each curve between 10 % of the first deviation from baseline following the response onset, and its return to baseline.

Results are expressed as mean ± SE of the mean. Standard deviation, when used, is indicated in the text. Significance was determined using paired *t*-test or ANOVA; *p* < 0.05 and the Bonferroni correction factor was applied. Normality was confirmed (Origin software) before using the paired *t*-test or ANOVA. *p*, *t,* and *F*(df_1_, df_2_) values are indicated in the text or figure legends.

#### *IN VIVO* RECORDINGS

Single-unit recordings were made *in vivo* in the IC of unanesthetized 1- to 2-month-old CBA/Ca mice. Mice were housed in rooms with reversed light-dark cycles and experiments were performed during the day, coinciding with their wake period. Data are reported from 109 cells in 32 animals.

#### Surgical procedures

Animals were anesthetized with isoflurane inhalation (1.5–2.0% in oxygen; Abbott Laboratories, North Chicago, IL, USA). The head of the animal was aligned in the stereotaxic apparatus at an angle of 20° (with some variations depending on age) to the horizontal. This head angle aligned the region of the midbrain containing the IC to be “flat,” in line with the rest of the brain so that the long axis of the IC was vertical. The hair on the dorsal surface of the animal’s head was removed, a midline incision made in the skin, and the underlying muscles reflected laterally to expose the skull. A metal pin was cemented onto the skull to secure the head to the stereotaxic apparatus used for recording, and a tungsten electrode cemented into the skull over the cerebrum for connection to ground. A small (~0.5 mm) opening was made in the skull to expose the dorsal surface of the IC. After surgery, a local anesthetic (Lidocaine) was applied to the surgical area, and the animal was allowed to recover. After each recording session, the craniotomy was covered with sterile bone wax to prevent damage to the brain.

#### Recording procedures

The animal was placed in a stereotaxic apparatus inside a single-walled sound chamber (Gretchken Industries, Lakeview, OR, USA) lined with polyurethane foam to reduce echoes. The head of the animal was positioned so that the IC was flat with respect to the horizontal and combined with a 90° fixed electrode impalement angle, allowing us to reach almost the whole range (~60 kHz) of characteristic frequencies in the IC (Egorova et al., 2006). Recordings on the same animal were performed for four consecutive days in two 2-h sessions each day, with a rest period of at least an hour between sessions. The animal was offered water from a medicine dropper between electrode penetrations. Signs of discomfort or distress were relieved either by light sedation with acepromazine (0.05 mg/kg) or by terminating the experiment.

Single-unit recordings were combined with pressure injection of HiDi. A glass recording electrode was glued to a five- or seven-barreled multi-pipette system (Havey and Caspary, 1980) pulled, broken and polished to a total tip diameter of <20 μm. The recording electrode was filled with 1 M NaCl (15–20 MΩ) or, in later recordings, with ACSF, and connected to a differential amplifier (A-M Systems). One barrel of the multi-barreled pipette was filled with 1 M NaCl or ACSF as a control for pressure injection. A second barrel contained HiDi, and the remaining barrels contained synaptic receptor antagonists dissolved in HiDi. Antagonists of inhibitory synaptic receptors, strychnine (8 {μ}M) to block glycine receptors and SR-95531 (Gabazine; 200 nM) to block GABA_A_ receptors, were dissolved in HiDi. These concentrations, which were higher than maximally effective concentrations in IC brain slices (Sivaramakrishnan et al., 2004; Chandrasekaran et al., 2013), were used to overcome inconsistent effects on firing rates because of variable diffusive loss caused by different pipette-cell distances in different recording sessions, and so that pressure ejection of the same drug from more than one pipette did not additionally affect firing rate. Gabazine and strychnine concentrations were varied in different experiments to examine their effects on depolarization block (Sivaramakrishnan et al., 2004) with and without HiDi. Recordings were tested for general recovery from drug applications by allowing recovery to occur following diffusive loss of the drug, or by application of normal ACSF or HiDi through another barrel. Complete recovery from strychnine was rapid (5–10 min); recovery from gabazine was slower (>30 min). These recovery times are similar to those in IC brain slices (Sivaramakrishnan and Oliver, 2006). In experiments not reported here, we also tested recovery from bicuculline. Recovery occurred rapidly, within 3–5 min, which is normal in brain slices, thus recovery times from strychnine and gabazine *in vivo* reflected their binding constants rather than artifacts of pressure injection. Chemicals were obtained from Sigma/Aldrich.

For pressure injection, the back end of a 1 ml plastic syringe was pulled to a fine tip, cut to the right length, inserted into each pipette, and glued at the end with Epoxy. The luer end of the syringe fitted into a needle, which was then inserted into pressure tubing (see **Figure 5A**). The five or seven tubes of the multibarrel electrode were connected to a picospritzer (WPI, Sarasota, FL, USA) through a set of valves that allowed independent control of each barrel. A second port on the picospritzer was connected to a vacuum inlet, which maintained a very low negative pressure (1–2 psi) on all barrels. Injection pressures were raised above vacuum pressures, and kept low (4–6 psi, 100–500 ms) to prevent cell damage.

#### Acoustic stimulation

Sound was delivered through a loudspeaker placed 10 cm in front of the animal at an angle of 15° to the midline, contralateral to the IC from which recordings were made. Acoustic stimuli were digitally synthesized and downloaded onto a digital signal processing card (AP2 Multi-Processor DSP card; Tucker-Davis Technologies, Alachua, FL, USA), converted to analog signals at a sampling rate of 500 kHz (model DA3-2; Tucker-Davis Technologies), filtered (model FT6-2; Tucker-Davis Technologies), attenuated (model PA4; Tucker-Davis Technologies), summed (model SM3; Tucker-Davis Technologies), amplified (model HCA-800II; Parasound, San Francisco, CA, USA), and sent to a loudspeaker (Infinity EMIT-B; Harmon International Industries, Woodbury, NY, USA). The output of the acoustic system was calibrated over a frequency range of 10–120 kHz using a condenser microphone (model 4135; Brüel and Kjaer, Nærum, Denmark) placed in a position normally occupied by the animal’s head. The calibration of speaker output at 0 dB attenuation was as follows: 4 kHz, 109 dB SPL; 40 kHz, 101 dB SPL; 50 kHz, 93 dB SPL; 80 kHz, 69 dB SPL. We needed to use a maximum tone frequency of 64 kHz, the upper limit of characteristic frequency (CFs). Harmonic distortion was not detectable 60 dB below the signal intensity using a fast Fourier analysis of the digitized microphone signal (model AD2; Tucker-Davis Technologies).

***Data acquisition and analysis.*** Custom software (Batlab; Dr. D. Gans, Northeast Ohio Medical University) was used to generate tone bursts, acquire spikes and frequency tuning curves, and display basic spike statistics in real time. Search stimuli consisted of tones, wide-band noise, and narrow-band noise bursts separated by 30–60 ms. Well-isolated single units were characterized by stable amplitude, consistent shape, and a signal-to-noise ratio exceeding 5:1. Once a single unit was isolated, its CF was determined. The CF was defined as the frequency at which the lowest sound pressure level consistently elicited stimulus-locked action potentials.

#### Construction and analysis of rate-intensity functions

Sound pressure level was increased systematically from 0 to 96 dB SPL in 5 or 10 dB increments for most recordings. In a few recordings (*n* = 8), we used randomly varying sound levels and did not observe significant differences in rate-intensity function (RIFs). Significance was determined with *t*-tests performed at five sound levels; *p* < 0.01. About 100 ms tone bursts were delivered at low rates (1/s) to prevent non-linearities in firing rate due to possible synaptic plasticity (Sivaramakrishnan et al., 2004). RIFs were generated at CF, using 12 repetitions at each sound pressure level. Tone onset was delayed for 300 ms following the onset of recording to allow for measurement of background firing rate. Background rates were averaged during the 300 ms window *prior* to the tone. For cells included in this study, the background firing rate introduced an additive constant to the RIF. To preserve a common denominator for RIF comparison across cells, background rates were subtracted from all RIFs in this study. Lack of background subtraction did not alter the results. Background rates were first examined for changes with sound intensity, and cells in which background firing rates changed were not included in the analysis.

Rate-intensity functions were constructed by averaging firing rates over the maximum response duration, measured from response onset, which was determined from the asymptote of first spike latency (FSL) plots. For each RIF, we first obtained the average firing rates and SD across all sweeps for each intensity. We then used *t*-tests to determine whether RIFs were significantly different in HiDi or drugs (*p* < 0.05). SD error bars are not included in illustrated graphs for clarity. Averages determined over other time windows, such as from the beginning of the sound stimulus or from the value of the median or lowest FSL, did not significantly alter the values of spike frequencies in this study. When comparing RIFs in different conditions, the maximum response duration was obtained from the group. Once steady state was reached, defined by little or no change in the RIF, we averaged three to four RIFs to obtain the steady state response.

#### Statistical tests

First spike latencies were calculated across 12 stimulus presentations, and reported as the minimum value of the median first-spike latency obtained across the sound levels tested in a RIF. We subtracted 0.3 ms to account for travel of sound across the 10 cm distance between the speaker and the animal’s ear and 0.5 ms for the rise time of the tone. Results are expressed as mean ± standard error of the mean. Standard deviation, when used, is indicated in the text. Normality was confirmed (Origin software) and paired *t*-test or ANOVA with *p* < 0.05, and Bonferroni correction, were used as a criterion for significance.

## RESULTS

In invertebrate systems and in acute mammalian brain slices, HiDi has been used to separate polysynaptic inputs from monosynaptically driven activity (Einum and Buchanan, 2004; Rose and Metherate, 2005). The effects of HiDi in blocking polysynaptic activity are generally attributed to an increase in firing threshold at the successive synapses in a string of synaptic contacts (Berry and Pentreath, 1976). High concentrations of divalent cations in the external bathing fluid can raise spike threshold by shifting sodium channel activation, generally observed at high (>3–4× normal) divalent concentrations (Campbell and Hille, 1976) or by local screening of ion channels that arises from the increased density of positive charges around their external surfaces (Gilbert and Ehrenstein, 1969; Hille et al., 1975).

To establish the effects of HiDi on intrinsic membrane properties as well as synaptic activity, and to find optimal divalent strengths for the IC, we tested the effects of HiDi in brain slices. We defined an optimal HiDi concentration in three ways. First, it should not result in intrinsic spike failure. This requirement was necessary to establish that HiDi did not prevent the postsynaptic cell from responding to a normally suprathreshold synaptic input. Second, HiDi should produce a clear separation between the synaptic components of an input. That is, to establish that the net synaptic input to a neuron contained a HiDi-sensitive and insensitive component, at least one of the two components had to have features that were invariant in HiDi. Third, under conditions of minimal stimulation of the ascending input fiber tract, when presumably only a few inputs were being activated (the ideal condition would be a single input), a single shock and a train of shocks should both evoke synaptic responses with the same onset latency. At identified synaptic contacts, minimal jitter in synaptic onset latency has been shown to be a more reliable indicator of a monosynaptic contact than either the absolute value of latency or the number of failures (Doyle and Andresen, 2001). A constant onset latency during stimulus trains would suggest that monosynaptic responses were retained in HiDi and further, that spike invasion of the nerve terminal was not compromised.

### HiDi EFFECTS IN IC BRAIN SLICES

As an assay for an optimal HiDi concentration, we first tested several concentrations on intrinsic membrane properties, and then established criteria for separating HiDi-sensitive and insensitive synaptic inputs. We found that raising Ca^+^^+^ and Mg^+^^+^ concentrations each by 2.5× normal was optimal based on criteria discussed in the following section. A 2.5-fold increase in Ca^+^^+^ and Mg^+^^+^ raised the total divalent concentration by 4.45 mM (2.5 HiDi; 5 mM CaCl_2_, 3.25 mM MgCl_2_; total divalent increase of 4.45 mM). This increase is modest compared with divalent concentrations used previously in mammalian brain slices (Einum and Buchanan, 2004; Rose and Metherate, 2005). We reduced external NaCl by an equivalent amount to maintain osmolality, which would have negligible effects on the total charge increased by the raised divalents.

#### HiDi effects on intrinsic membrane properties

**FIGURE 1:**
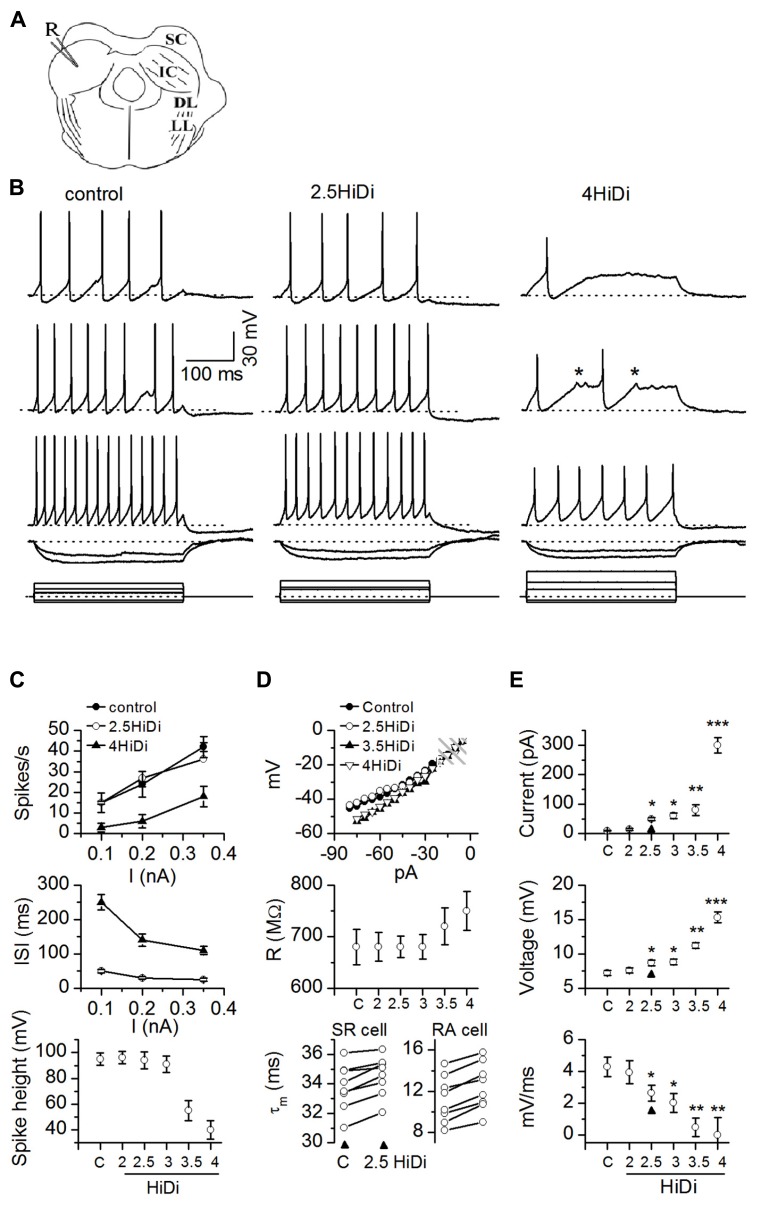
**HiDi concentration effects on intrinsic membrane properties.** **(A)** Transverse IC slice with patch electrode (R) for current injection and voltage recording. SC: superior colliculus; DL: dorsal nucleus of the lateral lemniscus; LL: lemniscal tract. Slices were bathed in different HiDi concentrations. **(B)** Intrinsic firing evoked by current steps. Sustained-regular cell (postnatal day 26). Left, middle: firing patterns remained sustained in 2.5 HiDi. Right: at higher divalent strengths (≥3.5 HiDi), spikes shortened and aborted (asterisks). When spiking did occur, firing remained sustained. Current steps are at the bottom of each column. Voltage responses are illustrated to three depolarizing and two hyperpolarizing current steps. Depolarizations increase from top to bottom; responses to the two hyperpolarizing currents are superimposed. Hyperpolarizing current steps were kept the same in ACSF and HiDi; depolarizing currents were increased in HiDi to evoke either roughly the same number of spikes as in the control (2.5 HiDi) or to cross firing threshold (4 HiDi). Currents were <0.5 nA to prevent membrane rupture, therefore spike frequencies in 4× HiDi did not reach control rates. **(C–E)** Summary of intrinsic membrane properties in control ACSF (C) and different HiDi concentrations. **(C)** Sustained-regular cells (P15–23). **(D)** Top, middle: sustained regular cells; Bottom: sustained-regular and rebound-adapting cells. **(E)** Data pooled from different intrinsic cell types. Cell type nomenclature from Sivaramakrishnan and Oliver (2001). **(C)** Spike characteristics. At ≤ HiDi, spike frequencies, inter-spike intervals, and spike heights were normal, ≥3 HiDi lowered spike frequencies, increased inter-spike intervals and decreased spike heights, 16 cells. Mean and SEM ANOVA for ACSF/2, 2.5, 3, 3.5, 4 HiDi. Spike frequencies: *F*_6,90_ = 2.82; *p* = 0.01; Inter-spike intervals: *F*_6,90_ = 2.87; *p* = 0.01. Spike heights: *F*_6,90_ = 3.01; *p* = 0.01. **(D)** Passive membrane properties in HiDi. Top: input resistance (R) plotted for one sustained-regular cell. Same cell as in **(B)**. Membrane potentials (ordinate) are relative to the resting membrane potential. Shaded area: the range of membrane potentials resistant to HiDi. Middle: average input resistances, 18 neurons. Mean and SD. Paired *t*-tests: ACSF, 2, 2.5, 3 HiDi, *p* > 0.33 in each condition. Input resistances deviated at larger hyperpolarizing currents; *p* < 0.004. Bottom: membrane time constants in control ACSF (C) and 2.5 HiDi shown for individual sustained regular cells (SR; left) and rebound-adapting (RA) cells (right). Eight cells in each panel. **(E)** Effects of HiDi on threshold. Top: injected current at which spike threshold was reached; Middle: membrane potential at threshold; Bottom: rate of rise of membrane voltage to threshold. Mean and SD 24 cells. Seven sustained-regular, 12 rebound, 5 pause-build. ^*^p < 0.01; ^*^^*^p < 0.001; ^*^^*^^*^p < 10^-^^5^.

Intrinsic membrane properties were examined by injecting current pulses into the soma and recording voltages with the same electrode (**Figure 1A**). Firing patterns evoked by depolarizing current steps were used to test the effects of HiDi on spike characteristics. Input resistances and membrane time constants were measured from responses to hyperpolarizing current steps. We report HiDi effects on sustained-regular cells (*n* = 14), a common IC cell type, chosen for its low spike thresholds, consistent spike heights during sustained firing, regular inter-spike intervals and negligible active conductances at small hyperpolarizations (Sivaramakrishnan and Oliver, 2001).

Firing patterns remained sustained in 2.5–3 HiDi (**Figure 1B**; left, middle). At higher divalent strengths (≥3.5 HiDi), spikes shortened and aborted (**Figure 1B**, right column). We therefore expected that divalent concentrations between 2.5 and 3× the normal would be optimal.

**Figures 1C–E** summarizes the effects of different HiDi concentrations on intrinsic membrane properties of sustained-regular cells. At ≤ HiDi, spike frequencies, interspike intervals, and spike heights were normal. At >3 HiDi, spike frequencies dropped, inter-spike intervals increased, and spike heights rapidly declined (**Figure 1C**; ANOVA, *p* < 0.05). These results suggested that 2.5 HiDi did not cause non-linear effects on voltage-gated conductances that underlie spiking in IC neurons, at least at the macroscopic level tested here. Non-linear effects at divalent strengths >3 HiDi are likely due, among other things, to high levels of charge screening or shifts in activation of voltage-gated sodium currents (Campbell and Hille, 1976).

About 2.5 HiDi did not alter passive membrane properties to significant extents. Input resistances and membrane time constants were measured with responses to small hyperpolarizing currents. Input resistances are illustrated for a single sustained-regular cell (**Figure 1D**; top) and averaged over 14 cells (**Figure 1D**, middle). Input resistances, measured at steady state, were normal up to ≤ HiDi (ACSF, 2, 2.5, 3 HiDi: *F*_4,68_ = 0.99; *p* = 0.42) but deviated at very large hyperpolarizing currents. Membrane potentials reached at these large hyperpolarizing currents were approximately -100 mV (30 mV hyperpolarization from a resting potential of -70 mV; **Figure 1D**, top and middle panels), which is out of the normal range of inhibitory synaptic potentials in IC neurons. If HiDi raises surface charge (Hille et al., 1975), the membrane would be expected to charge more slowly. In 2.5 HiDi, whole-cell time constants, derived from single exponential fits of voltage responses to small hyperpolarizing currents, increased by ~1–3 ms in different cells (**Figure 1D**, bottom panel), a very slight increase in the long time constants of IC neurons (11–37 ms in the different cell types; Sivaramakrishnan and Oliver, 2006). When averaged over the sample (*n* = 56; data pooled from different cell types), changes in time constant were minimized and were not significant in 2.5 HiDi (*t*_111_ = 1.53; *p* = 0.13), but showed significant differences at higher HiDi concentrations (e.g., 3.5 HiDi; *t*_111_ = 3.1; *p* = 0.002).

To examine the effect of HiDi on threshold for spiking, we measured threshold currents, voltages and the rate of rise of the membrane potential to threshold in response to current pulses injected into the soma. Threshold currents increased by 31 ± 8 pA in 2.5 HiDi (**Figure 1E**, top; arrowhead) and by 300 ± 69 pA in 4 HiDi (ACSF, 2 HiDi, *t*_27_ = 1.21; *p* = 0.23; ACSF, 2.5 HiDi, *t*_27_ = 3.3; *p* = 0.003; ACSF, 4 HiDi, *t*_27_ = 5.41; *p* < 0.0001). In 2.5 HiDi, threshold voltage increased by 1.1 ± 0.4 mV and the rate of rise of membrane voltage toward threshold slowed by 1.9 ± 0.5 mV/ms in (**Figure 1E**; middle, bottom). These data suggest that HiDi causes the membrane to charge more slowly, and raises firing threshold.

#### Identification of HiDi-sensitive and -insensitive components of synaptic responses

We next established the criteria necessary to distinguish a synaptic response as an extrinsic input that ascended through the lemniscal pathway and made monosynaptic contact on an IC neuron. Anatomical evidence suggests different origins for monosynaptic inputs to the IC central nucleus, arising from lemniscal afferents making direct glutamatergic (Loftus et al., 2004) or glycinergic (Moore et al., 1998; Loftus et al., 2004) contact onto neurons, or by corticofugal inputs (Nakamoto et al., 2013). Since corticofugal pathways were not accessible in slices, we restricted the interpretation of our data to lemniscal inputs.

**FIGURE 2:**
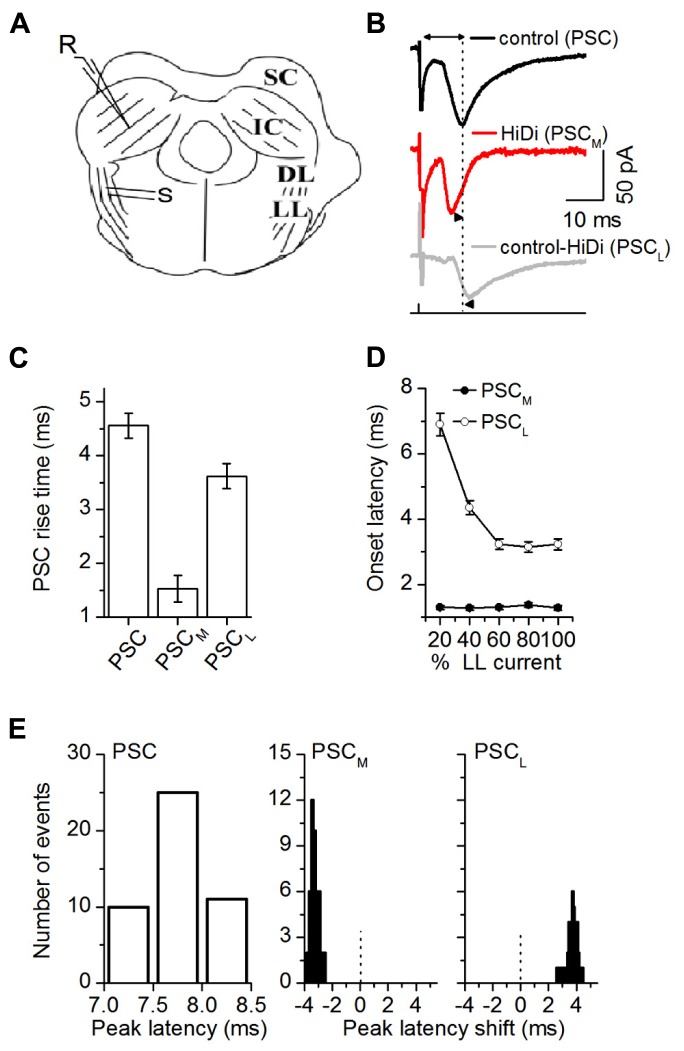
**HiDi isolates a monosynaptic component in brain slices.** **(A)** Transverse IC slice. SC: superior colliculus; DL: dorsal nucleus of the lateral lemniscus; LL: lemniscal tract. S: stimulation LL tract. R: recordings in the central region of the IC. **(B)** PSCs evoked by LL stimulus currents set at 50% above threshold levels. PSC: normal ACSF. PSC_M_: monosynaptic component of the PSC that remains in HiDi. PSC_L_ = PSC - PSC_M_; the local component blocked by HiDi. Double arrow, top trace: latency of the PSC peak measured from the stimulus artifact. Dotted vertical line is lined up to the peak of the PSC. Arrowheads, second and third traces: PSC_M_ and PSC_L_ peaks are shifted from PSC. **(C)** The rise time of PSC_M_ was faster than that of PSC_L_ (1.5 ± 0.2 and 3.6 ± 0.2 ms; *t*_23_ = 3.38; *p* = 0.0026). Rise times were measured between 10 and 90% of the rising phase, 24 cells. Mean and SEM. **(D)** Variation of PSC onset latency with LL stimulus current confirmed that PSC_M_ and PSC_L_ had monosynaptic and polysynaptic sources. The maximum stimulus current strength (100% LL current) is defined as the current beyond which no further increases in PSC amplitude or duration occur. PSC_M_ onset latencies had a SD <1, and remained constant as LL stimulus currents increased (Mean and SD: 1.31 ± 0.16 ms, *n* = 21 cells; 5 stimulus current strengths, *F*_5,100_ = 0.4; *p* = 0.84). PSC_L_ onset latencies shortened as stimulus currents increased (6.9 ± 0.4 to 3.23 ± 0.3 ms; *n* = 21 cells; *F*_5,100_ = 3.7; *p* = 0.004). Single LL shock. Onset latency was measured between the stimulus artifact and the time corresponding to a 10% deviation of the PSC from baseline, 21 cells. Mean and SD. **(E)** Distribution of the latencies and latency shifts of PSC peaks. Measurements of latency of the control PSC and latency shifts of PSC_M_ and PSC_L_ are illustrated in **(B)**. Left: absolute peak latencies of PSC; Middle, right: PSC peak latencies (7.76 ± 0.009 ms; *n* = 46 cells) were normally distributed. Peak latency distributions of both PSC_M_ and PSC_L_ were skewed. PSC_M_ preceded PSC by -3.32 ± 0.036 ms (*t*_179_ = 3.72; *p* = 0.0002), but PSC_L_ followed PSC by 3.78 ± 0.063 ms (*t*_179_ = 2.62; *p* = 0.009). Abscissa range of -3 to 1 indicates latencies relative to PSC, which is set at zero (dotted vertical lines). Gaussian fits *r*^2^ values, mean, and SEM PSC: 0.9974, 7.76 ± 0.009; PSC_M_: 0.8788, -3.3 ± 0.03; PSC_L_: 0.7369, 3.78 ± 0.06, 46 cells. **(B–E)** 2.5 HiDi.

We first confirmed that the PSC in ACSF contained ascending monosynaptic inputs. The afferent LL tract (**Figure 2A**) was stimulated with stimulus currents set at 50% above threshold levels to evoke PSCs. HiDi reduced PSCs, but only partially (**Figure 2B**), which suggested a HiDi-insensitive (monosynaptic, PSC_M_) and HiDi-sensitive (local, PSC_L_) component. The rise time of the HiDi-insensitive component was rapid (1.5 ± 0.2 ms; **Figure 2C**). The rise time of the total PSC (4.5 ± 0.23 ms), was less than the sum of the rise times of PSC_M_ and PSC_L_ (*p* = 0.0262) and implies conductance increases when both monosynaptic and local inputs were active. The onset latency of the HiDi-insensitive component, which was short (1.31 ± 0.16 ms, *n* = 21 cells), remained constant as stimulus currents were increased, with a SD <1 (Mean and SD: 1.31 ± 0.16 ms, *n* = 21 cells; five stimulus current strengths, *F*_5,100_ = 0.4; *p* = 0.84; **Figure 2D**). The constant onset latency, with a SD <1, which is a characteristic of identified monosynaptic cortical (Markram et al., 1997), thalamocortical (Rose and Metherate, 2005), and brainstem (Doyle and Andresen, 2001) inputs, suggested that the HiDi-insensitive component to the IC was a monosynaptic input, PSC_M_.

We next subtracted PSC_M_ from the total PSC, to obtain the HiDi-sensitive component. Since this component is the portion of the PSC that is not a direct ascending monosynaptic input, we refer to it as the local component, PSC_L_ (**Figure 2B**). The rise time of PSC_L_ was significantly slower than that of PSC_M_ (1.5 ± 0.2 and 3.6 ± 0.2 ms; *t*_23_ = 3.38; *p* = 0.0026; **Figure 2C**). Its onset latencies were much longer and shortened with stimulus intensity (6.9 ± 0.4 to 3.23 ± 0.3 ms; *n* = 21 cells; *F*_5,100_ = 3.7; *p* = 0.004). The decrease in onset latency with stimulus intensity is suggestive of a polysynaptic pathway. Given the placement of our stimulus electrodes on the lemniscal tract, the change in onset latency with stimulus intensity is likely due to a recruitment of multiple polysynaptic pathways onto a given IC neuron (See Discussion).

Of the total population of cells from which recordings were made (*n* = 166), 127 (76%) had both monosynaptic and local inputs, 11 (6%) had only monosynaptic inputs at all LL stimulus intensities, and in 28 neurons (17%), most or all responsiveness to LL stimulation was abolished by HiDi, suggesting a predominance of local polysynaptic inputs.

Because the HiDi-insensitive (monosynaptic) component did not exhibit changes in onset latency with stimulus intensity, it was unlikely to have included a significant fraction of the polysynaptic component. The largest separation of onset latencies between the HiDi-insensitive and sensitive components was 5.6 ms (1.3 and 6.9 ms) at the lowest lemniscal shock strength. This large separation suggests that a disynaptic input with an intermediate onset latency (Agmon and Connors, 1992) had not been activated. The separation times lessened to 1.94 ms (1.29 and 3.23 ms) at high shock strengths. This reduction in separation times could conceivably result from the activation of a high-threshold disynaptic pathway. The HiDi-insensitive component, however, did not increase its onset latency with stimulus strength (variations remained <1 SD), and did not fill in the 1.94 ms gap. The component isolated by HiDi was therefore less likely than the HiDi-sensitive component to have included the recruitment of a high-threshold disynaptic input.

Postsynaptic currents peak latencies (7.76 ± 0.009 ms; *n* = 46 cells) were normally distributed, but latency distributions of both the monosynaptic and local inputs were skewed (**Figure 2E**). The monosynaptic input preceded the total PSC (by -3.31 ± 0.036 ms; *t*_179_ = 3.72; *p* = 0.0002), but the local input followed it (by 3.78 ± 0.063 ms; *t*_179_ = 2.62; *p* = 0.009). The most likely interpretation of these early and late latencies is an electrotonic or spatial segregation of inputs on IC neurons or, given the variable extent of axonal collateralization in the IC (Oliver et al., 1991; Wallace et al., 2012), from sources with different path times.

**FIGURE 3:**
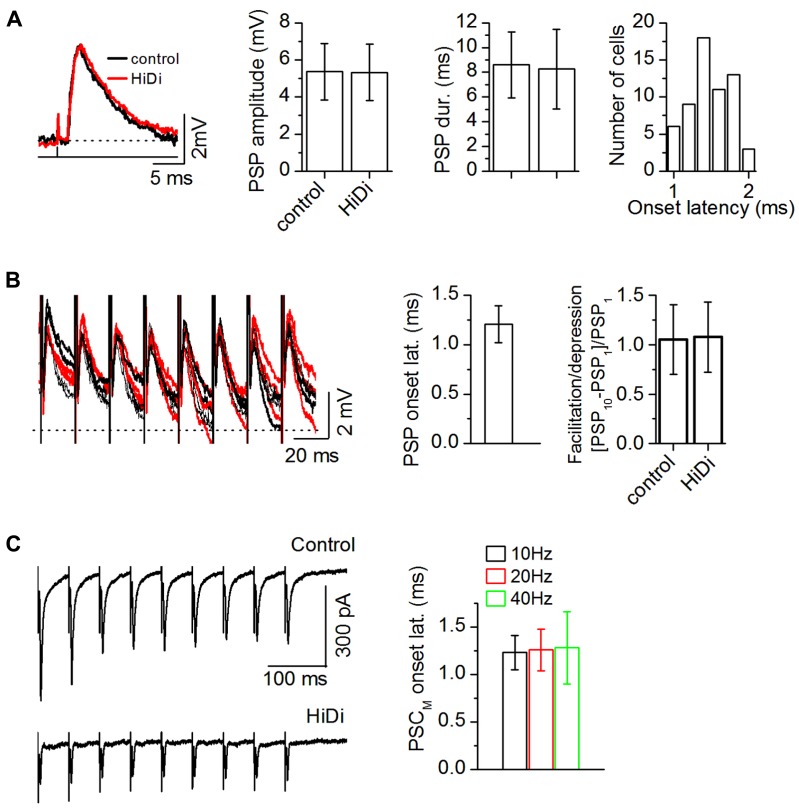
**Criteria for establishing a HiDi-insensitive response in slices as monosynaptic.** **(A)** HiDi does not affect postsynaptic potentials (PSPs) evoked by minimal (>50% failure rate) LL stimulation. Left: PSPs from one cell. Shock strength: 0.1 ms, 0.2 mA. Middle: PSP durations and peak amplitudes, 42 cells. Mean and SD. PSP duration: *t*_83_ = 0.71; *p* = 0.48; PSP amplitude: *t*_83_ = 0.48; *p* = 0.63. Right: the lack of variability in onset latency between trials confirmed a monosynaptic source. Distribution of onset latencies measured from the stimulus artifact, 60 neurons. Onset latencies were within a ~1 ms range (1.01–1.97 ms; mean 1.48 ms; SD 0.28). Because the SD of this distribution was <1 (0.28 ms), responses were considered monosynaptic if their onset latencies during repeated trials did not vary by >1 ms. **(B)** HiDi has no significant effect on PSPs evoked by a stimulus train at minimal shock strengths. Left: superimposed responses to repeated trials in one cell. Shock strength: 0.15 ms, 0.4 mA. PSPs remained insensitive to HiDi throughout the train (20–50 Hz trains; PSP amplitudes: *F*_9,290_ = 1.39; *p* = 0.19), 33 cells. Middle: PSP onset latencies during successive stimuli varied by less than one SD (1.21 ± 0.19 ms; 21 cells) verifying a monosynaptic input. Average of the first 5 pulses in 20 Hz trains of 10 pulses/train, 21 cells. Mean and SD. Right: ratio of the change in PSP peak amplitude between the first and last stimulus pulses, 20 Hz train, 10 pulses, 33 cells; Mean and SEM (*t*_65_ = 1.16; *p* = 0.25). **(C)** Left: recordings of synaptic currents evoked by a 20 Hz LL stimulus train with non-minimal (<10% failure rate) shock strengths (50% above threshold). Stimulus strength was adjusted to evoke a depressing response during the train. Holding potential -66 mV. Right: onset time of the HiDi-insensitive component (PSC_M_) during stimulus trains. Data are plotted for 10, 20, 40 Hz trains. Fifty-one cells were tested. Forty-two cells met the 1 ms SD criterion for onset variability. Mean and SD: 1.26 ± 0.22 ms. Data plotted from 25 of the 42 cells. **(A–C)** 2.5 HiDi.

As a more rigorous criterion for a monosynaptic response, we used minimal stimulation of the LL tract (50% synaptic failure rate) to confirm that the 1 ms onset latency established a boundary within which the HiDi-insensitive synaptic response could be considered to be monosynaptic. Minimal stimulation evoked PSPs that were completely insensitive to HiDi (**Figure 3A**; *n* = 42 cells; PSP duration: *t*_83_ = 0.71; *p* = 0.48; PSP amplitude: *t*_83_ = 0.48; *p* = 0.63). We presented single shocks at very low stimulus rates to get a sense of the variability in onset latency between trials (repetition rate of 1/3 s). This variability was within ~1 ms (1.01–1.97 ms; mean 1.48 ms; SD 0.28; *n* = 60 neurons). Because the SD of this distribution was <1 (0.28 ms), as was the SD of the HiDi-insensitive current evoked by different stimulus intensities and with trains of different frequencies (as in **Figure 2**), we concluded that the synaptic response insensitive to HiDi was a monosynaptic response. The tight clustering of onset latencies was surprising, because we expected that stimulation of the lemniscal tract would activate multiple inputs from various brainstem sources and that inputs would be distributed along dendrites, and onset latencies would vary, even within the short electrotonic distances that the somatic recording electrode would sample.

If there was considerable heterogeneity in the monosynaptic population either within release sites in a single monosynaptic input or within a group of monosynaptic inputs, then minimally evoked PSP amplitudes should vary during a stimulus train (Stevens and Wang, 1995). PSPs evoked by minimal-stimulus trains remained insensitive to HiDi throughout the train (**Figure 3B**; 33 cells; 20–50 Hz trains; PSP amplitudes: *F*_9,290_ = 1.39; *p* = 0.19). Onset latencies during the successive responses in the train varied by less than one SD (1.21 ± 0.19 ms; 21 cells). PSPs also did not facilitate or depress (*t*_65_ = 1.16; *p* = 0.25). We then raised the current strength of lemniscal stimulation to recruit more afferent axons, and switched to voltage-clamp to avoid postsynaptic non-linearities. The onset latencies of PSCs during stimulus trains varied by <1 SD (42/51 cells; **Figure 3C**; 25 cells; Mean and SDs: 10 Hz, 1.23 ± 0.18; 20 Hz, 1.26 ± 0.22 ms; 40 Hz, 1.28 ± 0.38). These data suggested constancy to the monosynaptic input to IC neurons, whether it was a single input or multiple inputs. A clustering of monosynaptic inputs close to the soma is one interpretation of our data, however, we suggest that, alternatively, the lack of variability in monosynaptic onset latency could arise from a dominant monosynaptic input (see Discussion).

**FIGURE 4:**
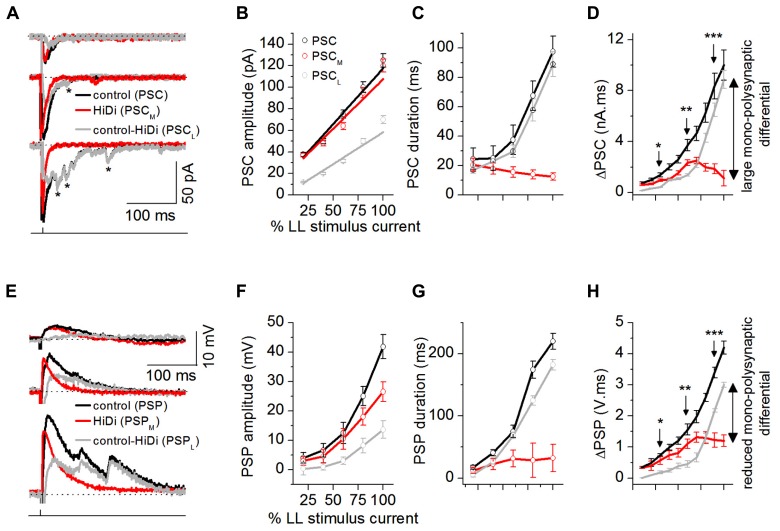
**Afferent input recruits distinct synaptic pools.** Dynamic ranges of the responses to mono- and polysynaptic inputs were measured by changing LL stimulus currents to evoke a minimal to maximal synaptic response (from 50% failures to a response that did not change with further stimulus increases). **(A)** PSCs evoked by changing LL shock strengths. Single LL shock: 0.1 ms, 1-2.5 mA. Top to bottom: 25, 50, and 80% of maximum shock strength. The maximum shock strength (100% LL stimulus current) is defined as the current beyond which no further increases in PSC amplitude or duration occur. Stimulus artifacts are truncated for clarity. Holding potential: -64 mV. Asterisks: long-latency synaptic currents. **(B–D)** PSC peak amplitudes, decay time constants, and integrals plotted as a fraction of the LL current that produced the maximum response (100%) for each cell. **(B)** Monosynaptic inputs controlled peak synaptic amplitude. PSC_M_ amplitudes were similar to PSC amplitudes at all stimulus currents (*F*_4,171_ = 1.72; *p* = 0.15), whereas PSC_L_ currents were smaller than PSC (*F*_4__,171_ = 4.14; *p* = 0.003), 35 cells. Mean and SEM. Linear fits; *r*^2^ values: PSC = 0.96339, PSC_M_ = 0.92598, PSC_L_ = 0.93378. **(C)** Polysynaptic inputs controlled synaptic duration. At low stimulus currents, PSC, PSC_M_, and PSC_L_ had similar durations (e.g., 20% LL current: *F*_2,93_ = 0.74; *p* = 0.48). With increases in stimulus current, PSC and PSC_L_ increased similarly in duration but PSC_M_ decreased in duration (e.g., 80% LL current: *F*_2,93_ = 14.6; *p* < 0.00001), 32 cells. Mean and SD, **(D)** PSC area integrals as a function of LL stimulus intensity, 32 cells. Mean and SD. ^*^F_2,94_ = 4.42; *p* = 0.015; ^*^^*^F_2,94_ = 6.34; *p* = 0.003; ^*^^*^^*^F_2,94_ = 7.22; *p* = 0.001. **(E–H)** HiDi effects on postsynaptic potentials. **(E)** PSPs evoked by changing LL shock strengths. Resting potential: -62 mV. **(F–H)** PSP peak amplitudes, durations, and integrals as a fraction of the maximum LL current **(F,G)** 35 cells. Mean and SEM, **(H)** 32 cells. Mean and SD, ^*^F_2,94_ = 4.06; *p* = 0.02; ^*^^*^F_2,94_ = 5.44; *p* = 0.006; ^*^^*^^*^F_2,94_ = 5.78; *p* = 0.004. Data are pooled from different intrinsic cell types (Sivaramakrishnan and Oliver, 2001). **(A–H)** 2.5 HiDi. Recording pipette contained QX-314. **(D,H)** Integrals of PSCs and PSPs were measured as the area under each curve between 10% of the first deviation from baseline following the response onset, and its return to baseline.

To get a sense of the “dynamic ranges” of monosynaptic and local inputs, we changed LL stimulus currents to evoke a minimal to maximal synaptic response (from 50% failures to a response that did not change with further stimulus increases; **Figure 4**). Recordings were made with QX-314 in the recording pipette to block sodium- and other voltage-gated currents (Mulle et al., 1985) and switched between voltage- and current-clamp to record PSCs and PSPs.

The PSC in ACSF increased with stimulus intensity and included smaller delayed events. PSC_M_ did not include the smaller events seen in the control PSC. As expected from temporal delays associated with polysynaptic inputs (Einum and Buchanan, 2005), the local input included long-latency events (**Figure 4A**). Peak PSC amplitudes were controlled by monosynaptic inputs (PSC, PSC_M_: *F*_4,171_ = 1.72; *p* = 0.15; PSC, PSC_L_: *F*_4,171_ = 4.14; *p* = 0.003; **Figure 4B**). PSC durations were more strongly affected by local inputs. At low stimulus currents, the total PSC, the mono- and local synaptic currents had similar durations (e.g., 20% LL current: *F*_2, 93_ = 0.74; *p* = 0.48). With increases in stimulus current, however, the local input increased in duration, and similarly to the PSC, but the duration of the monosynaptic current decreased (e.g., 80% LL current: *F*_2,93_ = 14.6; *p* < 0.00001; **Figure 4C**). The different trajectories of PSC_M_ and PSC_L_ durations with stimulus current verified that the smaller peak amplitudes of the local synaptic current were not due to just activity-dependent depression of the same synaptic pool (Waldeck et al., 2000) as the monosynaptic input.

Postsynaptic currents area integrals (ΔPSC) showed monosynaptic and local inputs with highest synaptic efficacies in adjacent stimulus current ranges. ΔPSC_M_ increased only until ~50% of the maximum LL stimulus current, after which it declined. ΔPSC_L_, which had an initially higher threshold than ΔPSC_M_ (>30% at 20% LL current), continued to increase with stimulus current. At peak LL currents, ΔPSC_L_ was 90% of ΔPSC, whereas ΔPSC_M_ was just 15% of ΔPSC, indicating a 75% monosynaptic-local input differential (**Figure 4D**).

The slow time constants of IC neurons (Sivaramakrishnan and Oliver, 2006) would be expected to favor integration of delayed local inputs. As stimulus current increased, the PSP, became multi-peaked and prolonged (**Figure 4E**). The monosynaptic component, PSP_M_, was single-peaked, while the local component, PSP_L_, was multi-peaked, reflecting integration of delayed local inputs. The monosynaptic-local input differential, seen with synaptic currents, was reduced, but not eliminated, by postsynaptic integration. PSP_M_ amplitudes reached just 63% of the PSP amplitude at 100% LL current, and durations increased only very slightly, saturating by 60% LL current strength (**Figure 4F**), suggesting active conductances (Sivaramakrishnan and Oliver, 2001). PSP_L_ amplitudes also did not reach those of PSP, however, their durations, which increased with stimulus current, were closely matched to the PSP duration (e.g., durations at 80% LL stimulus strength: PSC, PSC_L_: *t*_109_ = 1.21, *p* = 0.11; **Figure 4G**). PSP area integrals (ΔPSP) did not diverge as much as the corresponding ΔPSC values (**Figure 4H**; 40% PSP_L_ - PSP_M_ differential at 100% LL stimulus current). QX-314 blocks potassium currents and decreases their ability to rapidly repolarize PSPs in the IC (SS, unpublished observations). Our data therefore overestimates the capacity of the postsynaptic membrane to compensate for the divergence between mono- and polysynaptic inputs.

In summary, brain slice recordings established both an optimal divalent concentration of 2.5–3× the normal and, based on the rise times of synaptic currents, their onset latencies, the SD of latency distributions and the constancy of onset latency with stimulus intensity and repetitive stimulation, HiDi effectively separated monosynaptic inputs that ascend through the LL tract from local activity within the IC.

### HiDi EFFECTS *IN VIVO*

**FIGURE 5:**
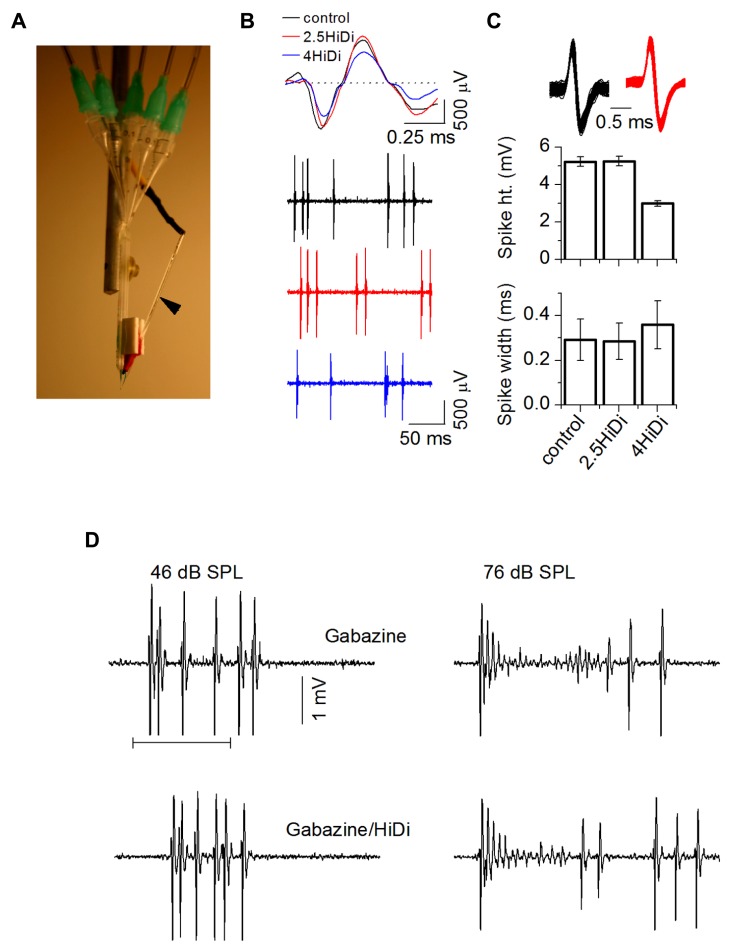
**Establishing optimal HiDi concentrations *in vivo*.** **(A)** Multi-barrel system for pressure injection and attached recording electrode (arrow). Pressure tubes are inserted into each barrel. **(B)** Spikes during single unit recordings were normal in 2.5 HiDi but shortened and broadened in 4 HiDi. Left: recordings of spiking in control, 2.5 HiDi and 4 HiDi. Top panel: superimposed traces of single tone-evoked spikes. Second through fourth traces: snippets of background activity in the absence of tones. The 50 ms scale bar applies to the second through fourth traces. **(C)** Quantification of HiDi-induced changes in spike heights and widths. Top: superimposed spikes from one neuron in control (black) and HiDi (red) illustrate that single unit isolation is unaffected by the pressure application of HiDi. Recordings illus{-}trated are at 81 dB SPL, suggesting that changes in spike rates in HiDi at high intensities were not due to false positives or false negatives. Bottom panels: spike heights and widths as a function of two HiDi concentrations. Mean and SD; Comparison between 60 spikes in control and 60 in each concentration of HiDi. Eight neurons. Spike heights: control, 2.5 HiDi: *t*_119_ = 1.01; *p* = 0.32; control, 4 HiDi: *t*_119_ = 2.56; *p* = 0.01. Spike widths: control, 2.5 HiDi: *t*_119_ = 1.2; *p* = 0.23; control, 4 HiDi: *t*_119_ = 3.01; *p* = 0.003). **(D)** Strong firing in HiDi does not produce spike loss due to additional depolarization block. Left and right columns show spikes at two intensities. Left: depolarization block at 46 dB SPL is not evident in either Gabazine alone or in HiDi/gabazine (left). The first spike latency in Gabazine alone occurs earlier than in HiDi, suggesting that some of the FSL is due to excitatory local circuits released from inhibition. Firing in HiDi is reduced, leaving the monosynaptic excitatory input. Right: depolarization block at 76 dB SPL is also observed in HiDi/Gabazine. HiDi does not produce additional depolarization block. 100 ms tone.

We used well-isolated (signal to noise >5:1) single unit recordings of neuronal discharge patterns in the IC of head-fixed unanesthetized mice to examine the effects of HiDi *in vivo*. To separate extrinsic monosynaptic inputs from local activity, HiDi was applied with pressure pulses for several minutes through one barrel of a multi-barrel electrode (**Figure 5A**). Each pulse was 500–1000 ms long and applied with pressures of 5–12 psi.

#### HiDi effects on spike characteristics

We performed control experiments to test for possible artifactual effects of HiDi *in vivo*. In 2.5 HiDi, single unit isolation was not affected and spike heights and widths were normal (*p* = 0.32; *p* = 0.226). Parallel to HiDi effects in slices, higher HiDi concentrations shortened and broadened spikes (**Figures 5B,C**). HiDi did not increase the likelihood of postsynaptic depolarization block that occurs in the IC at high sound intensities when inhibitory GABAergic input is blocked (Sivaramakrishnan et al., 2004). At sound intensities that did not elicit depolarization block in the GABA antagonist, gabazine, no block occurred in HiDi. At intensities that produced depolarization block in gabazine, HiDi evoked the same amount of block (*n* = 24; **Figure 5D**). This result suggests that 2.5 HiDi did not cause non-linear changes in the neuronal spike generator *in vivo*.

**FIGURE 6:**
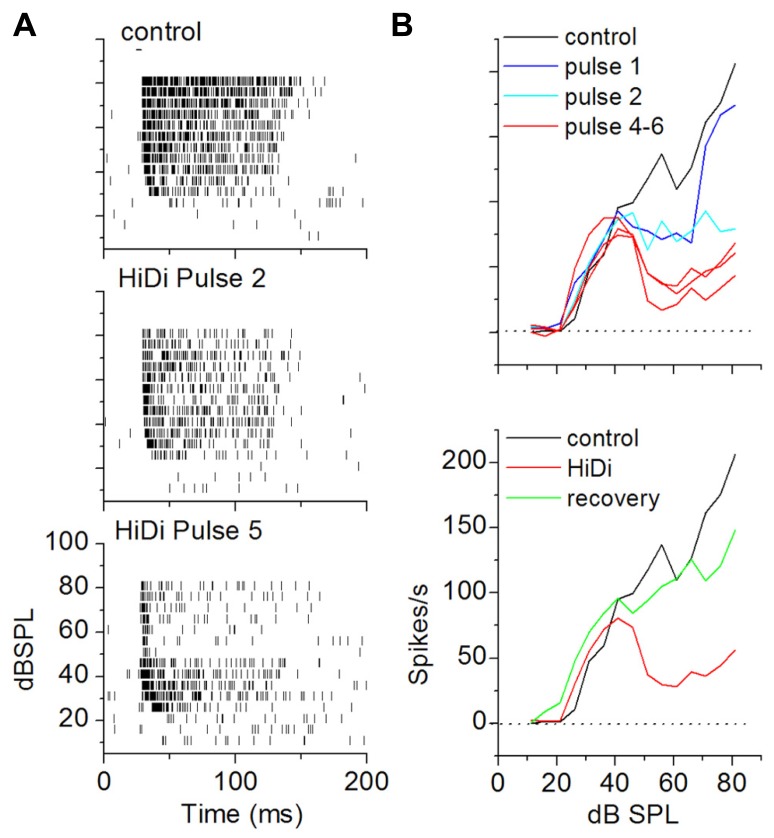
**Criterion for a complete separation of mono- and polysynaptic influences on firing rate *in vivo*.** **(A)** Achievement of steady state RIFs in HiDi. Raster plots in control (top) and after the second (middle) and fifth (bottom) pulse of 2.5 HiDi indicate gradually greater decrease in firing with successive HiDi pulses. **(B)** Top: change in the RIF with repeated HiDi application. In this cell, four HiDi pulses (each pulse was 500 ms, 8 psi) were required to reach a steady-state RIF. RIFs measured after the fourth, fifth and sixth HiDi pulses are superimposed. Bottom: recovery from HiDi (blue trace) ~6 min after the application was stopped.

The criterion for a complete separation of HiDi-sensitive and insensitive components of firing rate was a steady-state response in HiDi. To establish parameters for HiDi pressure injection that would produce steady-state responses, we constructed RIFs by varying tones between 0 and 90 dB SPL. This allowed us to test the effects of HiDi under conditions where the number of inputs and the range of frequencies recruited change with acoustic stimulation (Kiang et al., 1965). Since 2.5 HiDi did not produce spike shortening or depolarization block by itself, we were confident that changes in spike rates in HiDi could be attributed to changes in the relative complement of monosynaptic and local inputs, which would include the effects of postsynaptic integrating mechanisms. Steady-state firing rates were reached gradually through each subsequent HiDi pulse that produced intermittent changes (**Figure 6**). HiDi effects were reversible, which allowed us to record from several depths in the IC during one experiment. The reversibility of HiDi also suggested no long-lasting effects on membrane integrity.

#### Temporal activation of local circuits

**FIGURE 7:**
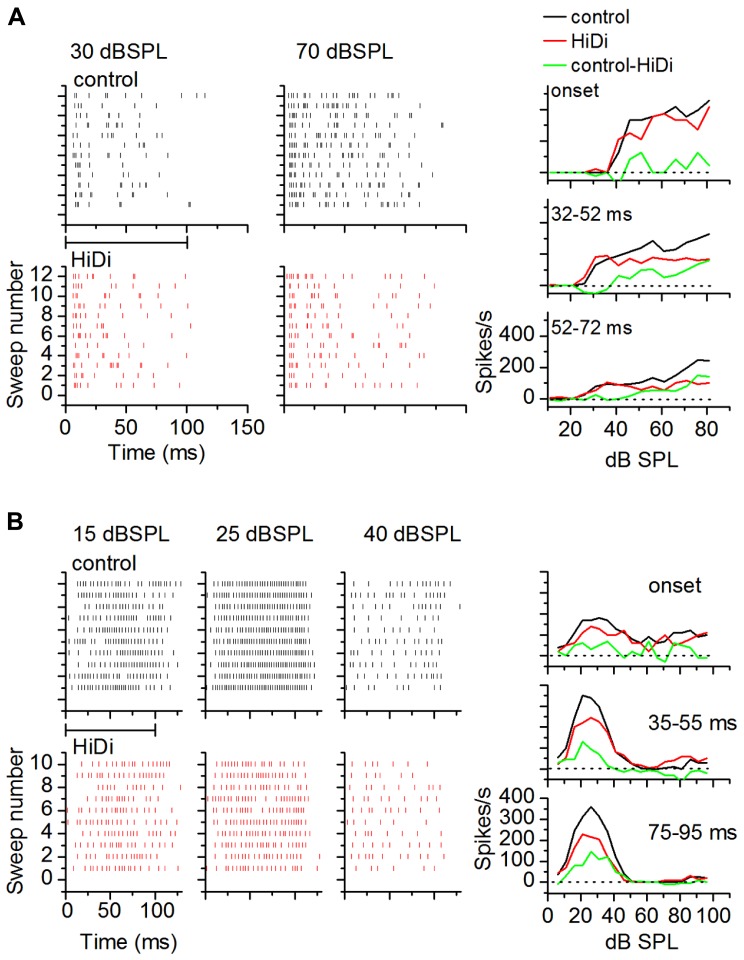
**Local circuits *in vivo* show temporal variations.** **(A,B)** Examples of HiDi effects on two neurons. **(A)** At 30 dB SPL, which was 15 dB above threshold for this neuron, HiDi did not affect spiking. At 70 dB SPL, HiDi reduced spiking, but mainly at later times during the tone. Rate-level functions plotted in different time windows during the tone for this cell (right column) show little effect of HiDi immediately following tone onset, but a larger effect ~20 ms after tone onset and 20 dB above threshold. **(B)** In a second neuron, HiDi does not affect firing at 15 dB SPL, but reduces firing at 25 dB SPL which corresponds to the peak of the control RIF (right column). HiDi has almost no effect at intensities corresponding to the downward limb of the RIF. HiDi does not affect responses soon after onset (top graph, right column), but its effect in successive time windows is similar.

To examine temporal variations in the HiDi-sensitive local component of the response, we measured the effects of HiDi on firing rates in successive time windows during a tone. **Figure 7** uses two cells to illustrate temporal variations in HiDi effects at different sound intensities. The cell in **Figure 7A** responded throughout the 100 ms tone. HiDi had no effect on firing rates at low sound intensities (e.g., 30 dB SPL), but decreased firing at high intensities (e.g., 70 dB SPL). At 70 dB SPL, the reduction in firing rate was most noticeable >50 ms after tone onset. The monosynaptic component overlapped the control RIF at very early times after tone onset (**Figure 7A**, right column, top panel), but contributed less at later times (**Figure 7A**, right column, middle, bottom panels). The firing rate due to the HiDi-sensitive local component was obtained by subtracting the monosynaptic RIF from the RIF in control conditions. This method of deriving local circuit effects on RIFs is simplistic, and assumes that the firing rate in HiDi includes interaction of local synaptic inputs with postsynaptic properties. It is used here merely to illustrate changes in firing rate in HiDi. The HiDi-sensitive component was not activated at tone onset, but increased at later times. In this cell, therefore, the local component turned on at 52 ms, and continued to increase with sound intensity. The cell in **Figure 7B** showed more temporally uniform HiDi effects. HiDi did not affect firing rates at low (15 dB SPL) and high (40 dB SPL) intensities. Its effect at 25 dB SPL, which corresponded to the peak firing rate of the control RIF, was distributed throughout the tone, with no particular distinction between tone onset and later times.

#### HiDi isolates monosynaptic inputs *in vivo*

**FIGURE 8:**
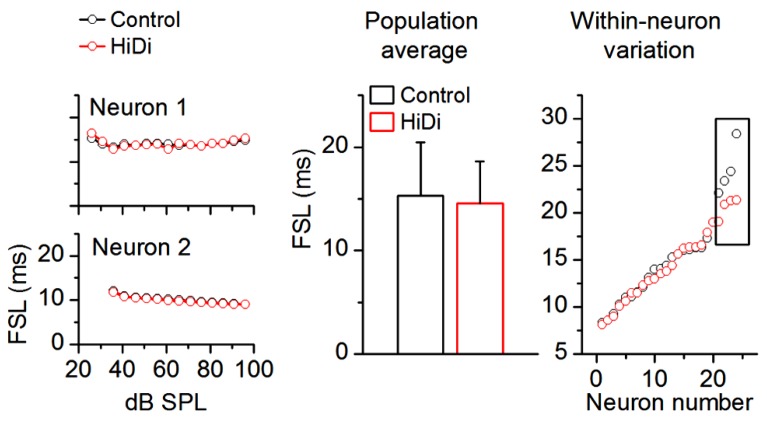
**HiDi does not affect most first spike latencies.** HiDi does not alter first spike latencies (FSL) in most neurons. Left: examples of FSLs in two neurons. Middle: population average, 82 neurons. Mean ± SD, *t*_163_ = 1.57; *p* = 0.118. Right: FSL distribution in 24 neurons. Each pair of data points is the FSL for a single cell. Boxed region: in a few cells with long FSLs (> ~25 ms), HiDi shortened onset latencies by 2–6 ms.

As a first requirement that extrinsic inputs to the IC would persist in HiDi, we tested its effects on first spike latencies (FSLs). Most FSLs in the IC arise from ascending input (Heil and Neubauer, 2001). FSLs involve direct monosynaptic pathways to the IC from the cochlear nucleus as well as ascending multi-synapse pathways (Mauger et al., 2010). We measured an average control FSL of 15.34 ± 6.15 ms SD (*n* = 88). The large SD of our population data reflects the wide distribution of FSLs (~5–30 ms), which has been previously reported in the unanesthetized IC (Sivaramakrishnan et al., 2004; Sanchez et al., 2007; Sayegh et al., 2012). If this wide range of FSLs reflects a range of monosynaptic, disynaptic, or polysynaptic inputs then, by parallels with brain slice recordings, a shortening of FSLs with increases in sound intensity should imply that a polysynaptic pathway within the IC was included in determining the FSL for a given neuron. Two aspects of the data in **Figure 8** suggest that most FSLs arose from monosynaptic inputs. First, in the two cells shown (**Figure 8**, left column), FSLs in HiDi did not shorten at high intensities (ANOVA, *p* < 0.05). This finding was consistent across the sample of IC neurons (60 neurons analyzed; ANOVA, *p* < 0.05) and suggested that the shortest latency input to an IC neuron was monosynaptic. Second, as an average in the population and in within-neuron comparisons, FSLs in HiDi were not altered (14.88 ± 6.03; 84/88 neurons; *p* = 0.118; **Figure 8**; middle, right panels). In four neurons with FSLs >25 ms, HiDi decreased FSLs by 2–6 ms. Local inhibition would be one source of long FSLs and could occur through recurrent inputs between IC neurons, and indicates that in a small population of IC neurons, FSLs involve local di- or polysynaptic connections. These data suggested that most FSLs in the IC arose from monosynaptic connections and further, that HiDi application did not compromise spike invasion into monosynaptic nerve terminals *in vivo*.

**FIGURE 9:**
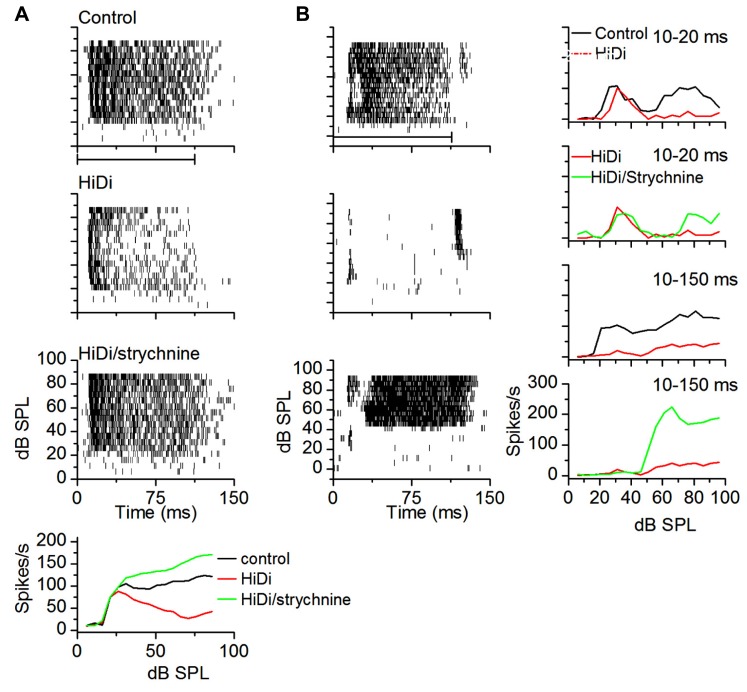
**HiDi does not block ascending monosynaptic glycinergic responses *in vivo*.** **(A)** Top three panels: spike rasters in control, 2.5 HiDi and 2.5 HiDi + 8 μM strychnine. Firing rates are reduced in HiDi but increased in HiDi/strychnine. The glycinergic input therefore inhibited firing rate in the presence of HiDi (the middle panel). The firing rate during the tone is due to a balance between (net) local excitation and ascending inhibition. Bottom: the RIF shows that the glycinergic input, which is an ascending inhibitory input, is not blocked by HiDi, thus HiDi preserves ascending lemniscal inputs. **(B)** A second example of a glycinergic input remaining active in the presence of HiDi. A neuron receiving ascending monosynaptic inhibition also receives local excitatory feedback. Left column: spike rasters in a cell with post-inhibitory rebound firing. HiDi did not abolish rebound firing but blocked activity during the tone. In strychnine/HiDi, firing during the tone returned and was strong, however, the threshold for firing was higher than in the control, suggesting that the glycine input was a high-threshold inhibitory input. Right: RIFs plotted early, 10–20 ms (from the first spike latency) and during the whole response to the tone (10–150 ms). At early times, the control RIF has two non-monotonic components that activate at low and high-intensities. HiDi blocks only the high-intensity component. Strychnine/HiDi, increased firing during the high-intensity component only. Thus this neuron receives two monosynaptic components: an excitatory input at low intensities and an inhibitory glycinergic input at high intensities.

Isolating monosynaptic inputs to the IC *in vivo* has not been simple because both inhibition and excitation ascend through the lemniscal pathway. Using high local levels of GABAergic agonists raises the threshold for local excitation (Zhang and Kelly, 2003) however it is not clear what the suppressive effect would be on ascending excitation. To test whether we could use HiDi to isolate monosynaptic input to the IC, we tested its effects on glycinergic inputs, which are well-established as being monosynaptic (Moore et al., 1998; Loftus et al., 2004). We measured firing rates in HiDi, and again in a HiDi/strychnine combination. Firing rates in HiDi/strychnine increased beyond control rates. This increase was observed in a number of neurons with different patterns of glycinergic input (*n* = 17; **Figure 9**) and provided one piece of evidence that we could use HiDi to isolate monosynaptic inputs *in vivo*. We chose the two cells in **Figure 9** to illustrate that the preservation of monosynaptic inputs by HiDi is not necessarily a reflection of their strengths. The neuron in **Figure 9A** retained sustained monosynaptic excitation during the tone (**Figure 9A**, middle panel), thus glycinergic inhibition was not strong enough to completely block monosynaptic excitation. Glycinergic input did decrease monosynaptic excitation, however, since the firing rate in HiDi/strychnine was greater than in HiDi alone. In the neuron in **Figure 9B**, glycinergic inhibition was strong enough to completely block the monosynaptic excitatory input. Had the balance been toward monosynaptic excitation, part of the firing observed in HiDi/strychnine should have occurred during the tone in HiDi alone.

## DISCUSSION

The ability to isolate inherited inputs from local circuitry allows a dissection of hierarchical processing in systems. Our results suggest that the use of HiDi *in vivo* succeeds in isolating the monosynaptic component of the response to an acoustic stimulus. Because we applied HiDi locally, the component that is sensitive to HiDi is most likely a local component. Although parallels between brain slices and *in vivo* recordings are subject to interpretation, our measurement of HiDi effects *in vitro* and *in vivo* provide strong evidence that HiDi distinguishes at least two synaptic pools, one a monosynaptic pool ascending through the lemniscal pathway into the auditory midbrain, and a second pool that consists of local di- and polysynaptic pathways.

The component of HiDi-insensitive responses in the IC evoked by electrical stimulation of the lateral lemniscus or by acoustic input appears to have the characteristics of a monosynaptic input. Its onset latency shows little jitter during repeated activation of inputs, and it gives rise to the FSL *in vivo*. Increasing recruitment of lemniscal inputs *in vitro* or increasing the tone duration recruits additional inputs that are HiDi-sensitive and change their onset latencies with intensity, suggesting a polysynaptic effect of local IC connections. While the limited jitter in onset latency of the synaptic response characterizes identified monosynaptic inputs *in vitro* (Markram et al., 1997; Doyle and Andresen, 2001; Rose and Metherate, 2005), we cannot rule out the possibility that disynaptic components, whose reliability could increase through factors such as spike-timing dependent plasticity (Babadi and Abbott, 2010), comprise some part of the HiDi-insensitive response.

### SOURCES OF MONOSYNAPTIC AND LOCAL INPUTS

The amplitude of the rapidly rising, short latency, monosynaptic current in brain slices, determined the amplitude of the total synaptic current, but it also reached its peak earlier, suggesting large glutamatergic terminals or dense terminal arbors (Winer, 2005; Nakamoto et al., 2013) on proximal dendrites. The concentric bipolar electrode that we used to activate lemniscal axons would have restricted current spread to some extent at low current strengths. However, with a 100 μm active tip, several lemniscal axons would have been activated. Low stimulus currents would have activated the largest diameter axons and those closest to the stimulating electrode surface. These inputs, if monosynaptic, would have created the shortest onset latencies that we measured. Increases in current strength and the gradual recruitment of axons in order of large to small diameter would add to the monosynaptic latency. Our results, however, did not show a change in onset latency >1SD. Therefore, either monosynaptic inputs are tightly clustered on proximal dendrites, or each IC neuron receives a single predominant large axon input from the lemniscus, which provides a dominant onset latency. Rise times, which would have included summation of additional recruited monosynaptic inputs, were ~1.3 ms. If there was a monosynaptic population arising from axons of different diameters giving rise to the 1.3 ms rise time, the absolute value of the rise time could in some part be a measure of how close the stimulating electrode was to the IC. *In vivo*, effects of differences in axon diameter would be magnified by distance from the IC, so that there would be a greater spread in rise times with recruitment and summation of monosynaptic inputs.

*In vivo*, most FSLs did not change in HiDi, also suggesting a dominant monosynaptic lemniscal input to each neuron. Unlike the slice recordings, however, FSLs were widely distributed in IC neurons 5–30 ms, strongly suggesting that inputs to different IC neurons arose from axons with different diameters (Malmierca et al., 2005), conduction velocities or path distances. The few long latency FSLs, which were shortened in HiDi, could imply local inhibitory influence.

The decrease in onset latency of HiDi-sensitive local inputs suggests that increased stimulus intensity at the lemniscus recruits multiple local pathways whose final synapses impinge on the dendrites of an IC neuron at different distances from the soma, or that summation of different local inputs produces a response that reaches the soma faster. The distal dendritic location of local input suggested by the delayed response in brain slices could arise from axonal collateralizations of IC neurons within frequency laminae or more widespread (Oliver et al., 1991; Wallace et al., 2012) local inter-neuronal connections. Polysynaptic activity generates plateau potentials in the IC and elsewhere (Rose and Metherate, 2005; Sivaramakrishnan and Oliver, 2006; Takahashi and Magee, 2009). These local synapses would therefore be focal targets of HiDi. NMDARs activate close to the resting potential in a substantial population of IC neurons (Wu et al., 2004; Sivaramakrishnan and Oliver, 2006) and local circuit regulation of dendritic excitability involving glutamate receptors or voltage-gated channels (Yu and Salter, 1999; Ohtsuki et al., 2012; Lee et al., 2013) would be expected to provide an increased gain associated with large and prolonged local potentials. The longer, slower polysynaptic responses most likely reflect the fact that local inputs are less synchronously activated. The long pathways involved in commissural influences on local processing (Orton et al., 2012) or the more spatially restricted dendritic arbors within anatomical laminae (Morest and Oliver, 1984; Oliver and Morest, 1984; Serviere et al., 1984; Brown et al., 1997) could underlie the variability in the temporal activation of local inputs.

In summary, high concentrations of divalent cations, which have been used to separate monosynaptic inputs from local circuits in invertebrates and mammalian brain slices, appears to be a viable tool to isolate monosynaptic extrinsic inputs from local circuits *in vivo*.

## Conflict of Interest Statement

The authors declare that the research was conducted in the absence of any commercial or financial relationships that could be construed as a potential conflict of interest.

## AUTHORS CONTRIBUTION

Shobhana Sivaramakrishnan performed slice experiments. Calum Alex Grimsley, Jason Tait Sanchez, and Shobhana Sivaramakrishnan collected *in vivo* data. Calum Alex Grimsley and Shobhana Sivaramakrishnan analyzed *in vivo* data. Shobhana Sivaramakrishnan designed the study and wrote the paper.
